# Novel gastrointestinal tools (GI Tools) for evaluating gut functional capacity in adults with environmental enteropathy in Zambia and Zimbabwe: A cross-sectional study protocol

**DOI:** 10.12688/f1000research.154471.1

**Published:** 2024-08-23

**Authors:** Tracy N. Phiri, James W. Weatherill, Elena Monford-Sanchez, Jose-Ivan Serrano-Contreras, Callum Melvin, Mirriam Kunaka, Ian Chisenga, Perpetual Ngalande, Monica N. Mweetwa, Ellen Besa, Tafhima Haider, Nilanjan Mandal, Alex J. Thompson, Christine A. Edwards, Claire D. Bourke, Ruairi C. Robertson, Joram M. Posma, Rosemary Banda, Mulima Mwiinga, Lydia Kazhila, Leolin Katsidzira, Mutsa Bwakura-Dangarembizi, Beatrice Amadi, Isabel Garcia-Perez, Kathryn Maitland, Julian R. Marchesi, Douglas J. Morrison, Gary Frost, Paul Kelly

**Affiliations:** 1 Tropical Gastroenterology & Nutrition group, University of Zambia School of Medicine, Lusaka, Lusaka Province, Zambia; 2 Stable Isotope Biochemistry, Scottish Universities Environmental Research Centre, East Kilbride, Scotland, G75 0QF, UK; 3 Imperial College London Faculty of Medicine, London, England, W12 0NN, UK; 4 Department of Metabolism, Digestion and Reproduction, Imperial College London Section of Nutrition Research, London, England, W12 0NN, UK; 5 School of Medicine Dentistry and Nursing, University of Glasgow College of Medical Veterinary and Life Sciences, Glasgow, Scotland, UK; 6 Blizard Institute, Queen Mary University of London Barts and The London School of Medicine and Dentistry, London, England, E1 2AT, UK; 7 Centre for Immunobiology, University of Glasgow College of Medical Veterinary and Life Sciences, Glasgow, Scotland, UK; 8 Zvitambo Institute for Maternal and Child Health Research, Harare, Harare Province, Zimbabwe; 9 Department of Internal Medicine, University of Zimbabwe College of Health Sciences, Harare, Harare Province, Zimbabwe; 10 Department of Child, Adolescent and Women’s Health, University of Zimbabwe College of Health Sciences, Harare, Harare Province, Zimbabwe; 11 Department of Infectious Disease and Institute of Global Health and Innovation, Imperial College London Faculty of Medicine, London, England, UK

**Keywords:** Environmental Enteric Dysfunction, Intestinal Absorption, Intestinal Permeability, Microbiome, Stable Isotopes

## Abstract

**Background:**

Environmental enteropathy (EE) is a highly prevalent subclinical inflammatory intestinal disorder associated with growth failure, impaired neurocognitive development, poor response to oral vaccines, and micronutrient deficiencies. However, EE research and clinical trials are hampered by the lack of non-invasive tools for measuring intestinal function in detail. This study aims to develop new tools for the measurement of multiple domains of gut functional capacity.

**Methods:**

The GI TOOLS project is a cross-sectional study that will recruit adults aged 18-65 years with EE in Lusaka, Zambia. Each participant will undergo assessment of gut functional capacity using novel near-point-of-care tools and provide multiple samples for detailed laboratory analyses. Participants will also undergo endoscopy for collection of duodenal biopsies. Novel techniques include stable isotopes approaches to measuring digestion, absorption, and bidirectional transmucosal amino acid flux, a non-invasive fluorescence tool for real-time evaluation of gut permeability, and assessment of reverse permeation of intravenous antibiotics to be carried out separately in Zimbabwe. Stool and duodenal microbiome sequencing using MinION sequencing, metabolome analysis applied to plasma and intestinal fluids, blood immune cell phenotyping, *in vitro* epithelial barrier models, and duodenal immunohistochemistry will also be used to explore EE in depth. These will all be integrated with gold standard histology and mucosal morphometry, alongside lactulose permeation data, and stool and plasma biomarker analysis. The protocol has been approved by ethics committees and regulators in Zambia, Zimbabwe, and the UK. Participants will give informed consent before they can participate

**Anticipated outcomes:**

Based on this extensive phenotyping, tests will be developed which can be simplified and refined for use in adults and children with EE, and for clinical trials. Findings from this project will be disseminated through in-person meetings with caregivers and regulatory bodies, presentations at conferences and in peer-reviewed journals.

## Introduction

The gastrointestinal tract is a critical organ, simultaneously orchestrating digestion, absorption, and excretion of gut contents whilst providing a physical barrier between the external environment in the gut lumen and body tissues, creating conditions to tolerate dietary antigens and the gut microbiome. ^1^ However, measurement of these intestinal functions, which we refer to hereafter as gut functional capacity, is currently crude and imprecise. There is an urgent need to develop improved tools for the evaluation of a broader range of intestinal functions in studies of pathophysiology and clinical trials in both adults and children. ^1^ Following a golden age of research on human intestinal digestion and absorption in the 1970s and 1980s, work on gut physiology has slowed. Current gold-standard approaches to assess gut functional capacity, such as the lactulose-mannitol (or lactulose-rhamnose) test or endoscopy, are time- and labour-intensive or measure only one aspect of intestinal dysfunction, such as its permeability to molecules which normally are excluded from uptake in the healthy state.

Environmental enteropathy (EE) is a highly prevalent subclinical inflammatory intestinal disorder that is associated with exposure to unsanitary conditions and poor nutrition. In children, it is associated with growth failure, impaired neurocognitive development, and poor response to oral vaccines. EE is frequently associated with undernutrition. In moderate undernutrition, limited nutrient bioavailability, pathogen pressure, and gut barrier dysfunction can lead to impaired linear growth (stunting), impaired vaccine response and cognitive deficits in children. ^2^ In acute undernutrition, mortality remains unacceptably high, particularly those hospitalised with severe acute malnutrition (SAM). ^3^^,^^4^ Both stunting and incomplete recovery from SAM in childhood embed life-long and intergenerational health consequences which detrimentally impact population and economic health in low- and middle-income countries (LMICs). ^5^^–^^7^

**Figure 1.**
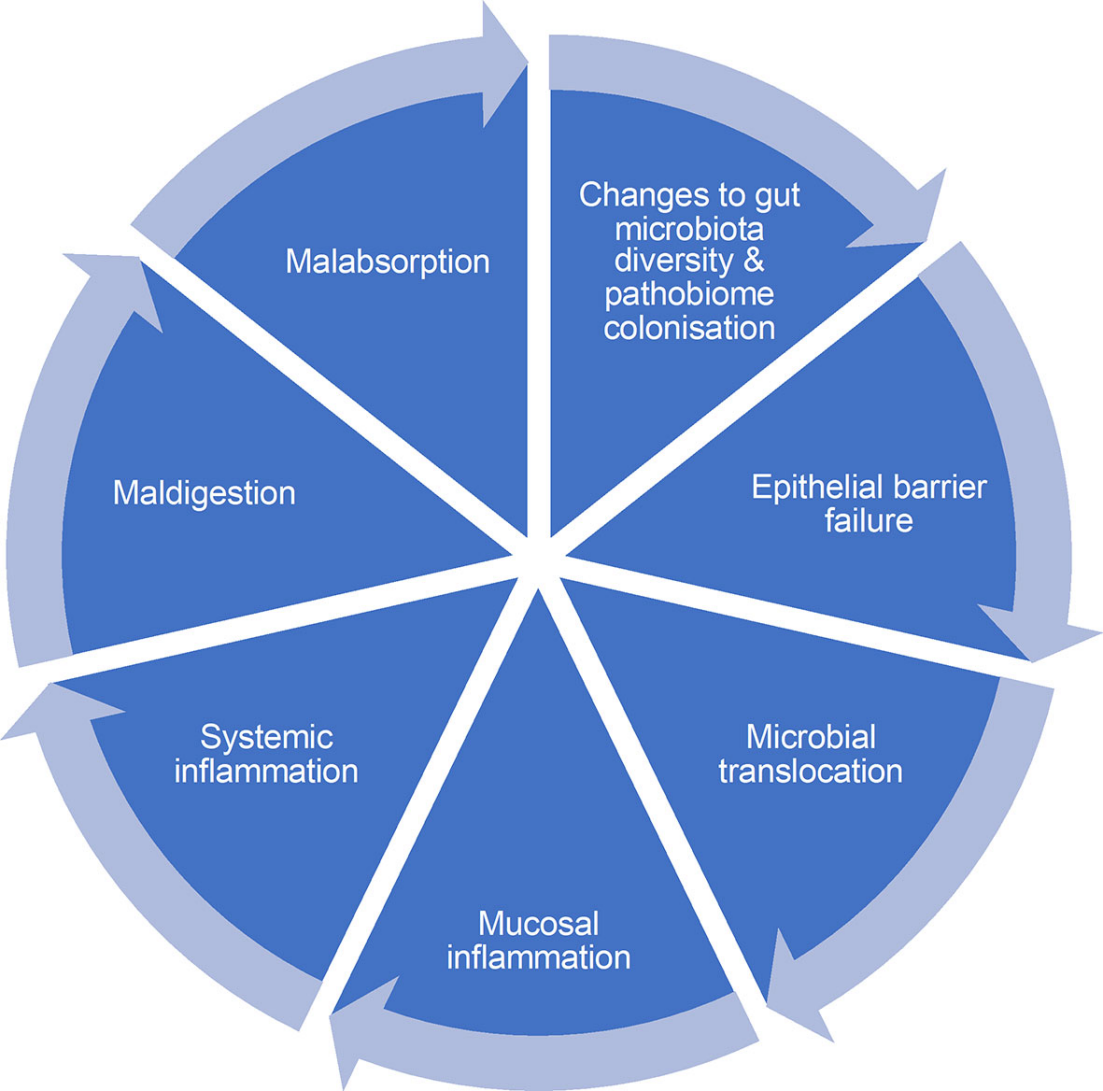
Domains of intestinal dysfunction leading to cycle of inflammation and persistence. Illustration of prominent elements of inflammation cycle, that includes (i) changes to gut microbiota diversity and pathobiome colonisation leading to decompartmentalization, which promotes (ii) epithelial barrier failure leading to increased permeability and (iii) microbial translocation of cell wall antigens and bacteria, which stimulates (iv) mucosal inflammation leading to (v) systemic inflammation which leads to increased demands for essential nutrients (e.g. amino acids), whose bioavailability may be limited by (vi) maldigestion caused by alterations to brush border architecture and enzyme activity and (vii) malabsorption caused by changes to the expression of nutrient transport proteins in epithelial cells. At the present time, it is not possible to determine how this network of functions is interrelated, and which functions are critical to resolve without new tools to assess each of these domains.

EE probably represents a chronic adaptation of the proximal small intestine to marginal diets and exposure to environmental enteropathogens. ^8^ In LMICs, this adaptation develops in early life and is characterised by reduced absorptive surface area, goblet and Paneth cell depletion and intraepithelial lymphocyte infiltration. ^9^ Our work indicates that SAM is associated with a more severe enteropathy ^10^; a global disturbance of intestinal architecture and function, including maldigestion, malabsorption and impaired gut barrier function. ^13^ Impaired gut barrier function allows translocation of microbes and their products, which may explain systemic endotoxemia and, in some cases, sepsis and septic shock, which are major drivers of mortality. ^11^^–^^13^ Both SAM and the antibiotic treatments recommended for children admitted to hospital with SAM also affect the gut microbiome, the composition of which is associated with lower bacterial diversity and maturity, which fail to persistently recover with standard nutritional therapies. ^14^ There is a need to take a holistic approach to understanding the impact of undernutrition and EE on gut physiology across the whole gastrointestinal tract, which means developing tools for assessing multiple domains ^15^ of gut function ( Figure 1). This necessitates simultaneous assessment of the structure of the intestine (e.g. villus height, and therefore surface area), barrier function and microbial translocation, digestive and absorptive capacity (e.g., enzyme activity for protein, fat, and carbohydrate digestion, transporter expression for absorption), systemic, and intestinal immune responses to pathogens, expression of epithelial pattern recognition receptors (PRRs), pathogen-associated molecular patterns (PAMP; an indicator of microbial translocation), and the composition, function and metabolic activity of the gut microbiota. The latter will be reflected in the measured host metabolome. Such measurements will allow the selection of an optimised ‘toolkit’ for assessing gut functional capacity and ultimately enable the design of novel therapeutic feeds, which actively promote improved gut function, support sustained rehabilitation, reduce mortality, and feed the superorganism (i.e., the host and microbiome), and not just the host. Recent evidence has demonstrated that designing a diet to enhance normal maturation of the colonic microbiota results in improved growth ^14^^,^^16^^,^^17^ versus standard therapies in children recovering from acute malnutrition. Probiotic interventions have the potential to improve recovery and growth in various malnutrition disorders, ^18^ providing the proof-of-principle that rational design of feeds or biotherapeutics targeted to support gut function could be an achievable and effective therapeutic approach at scale.

The urgent problem which we aim to address in the GI Tools study is the dearth of measurement tools to assess the gut functional capacity of communities affected by EE. These tools will 1) be essential for evaluating gut functional capacity in adults (across many disease states) 2) have the potential to assess gut functional capacity in children because of their largely non-invasive nature, and 3) direct the development of new therapeutic interventions to support optimal gut function, for example, recovery of children hospitalised with SAM.

## Aims and objectives

The overarching objective of the GI Tools project is to develop a novel portfolio of tools for evaluating gut functional capacity in EE and malnutrition disorders. This protocol will allow for the determination of safety, acceptability, dosing, and timing for novel tools to evaluate gut functional capacity in adults with EE. This will be translated to work in malnourished children in further phases of this work.

### Study aims

Aim 1: Evaluate a novel portfolio of stable isotope-based tools for evaluating carbohydrate and protein digestion and amino acid absorption.

Aim 2: Test a novel non-invasive fluorescence sensor-based tool for real-time evaluation of gut permeability.

Aim 3: Characterise the microbiome and metabolome across well characterised and novel physiological sites (duodenal aspirate, duodenal mucus, duodenal tissue, stool, plasma, urine).

Aim 4: Quantify systemic and intestinal inflammation using established biomarkers of EE, immune cell phenotyping, epithelial PRR expression, and in vitro epithelial barrier models.

Aim 5: Determine if antibiotics, given intravenously, permeate into the gut lumen in sufficient quantities to modulate the microbiota.

All of these will be correlated with histological mucosal morphometry, lactulose permeation, and established biomarkers of EE.

### Study strengths and limitations

• A study of 80 adults with EE will develop novel analytical tools to demonstrate their viability and effectiveness for the assessment of gut functional capacity compared with current ‘gold standards’.
• This study will inform future work on EE disease assessment and in treating children with severe acute malnutrition (SAM) and/or stunting.
• These novel tools will have practical applications in clinical assessments not only in EE but other conditions that affect gut functional capacity allowing better understanding of the underlying mechanisms of different diseases.
• A limitation of this study is the lack of country-specific controls in whom the same assays would be performed for comparison.
• Including adults only in this study limits the applicability of some of the analytical methods when designing a child-based study due to smaller sample volumes.

## Methods

### Study outcomes

The expected outcomes for each aim are shown in Table 1.

**Table 1.** Summary of outcomes with reference to study aims.

	Measured outcome	Detail
*Aim 1*	*Protein digestion and absorption using stable isotope tracers*	Assessment of ^13^C spirulina digestion (global index by breath ^13^CO _2_) and absorption using ^13^C Phe (from spirulina) and ^2^H labelled free Phe administered orally.
	*Bidirectional transmucosal amino acid flux (BTAAF)*	A novel tool involving dual infusion of differentially labelled amino acid tracers (Phe/Leu) orally and intravenously for the assessment of EE severity.
*Aim 2*	*Fluorescein*	A novel non-invasive measure of intestinal permeability using orally dosed fluorescein and a finger probe (transcutaneous fluorescence spectroscopy) for detection of systemically circulating fluorescein.
*Aim 3*	*Microbiome analysis*	I. Faecal species abundance and diversity indexes by 16s rRNA sequencing. II. In country deep metagenomic sequencing by long read (Nanopore) sequencing compared with other short-read platforms (Illumina). III. Evaluation of microbial communities in EE and their ability to uptake intestinal amino acids, including conversion of L-amino acids to D-forms.
	*Metabolic phenotyping*	I. Untargeted metabolomics ( ^1^H-NMR) of plasma, urine and faecal water. II. Targeted quantification (MS) of intestinal, plasma and urine amino acids (unlabelled + labelled) before and after administration of oral and intravenous stable isotopes.
	*Metaproteomic analysis*	1) Proteomic analysis of intestinal biopsies for expression of key mucosal proteins in biopsies (including mucins). 2) Dynamic proteomic analysis of key target proteins to assess mucosal protein synthesis rates in biopsies by high resolution MS and D _2_O labelling.
*Aim 4*	*Biomarker analysis*	Quantification of known markers of systemic inflammation, microbial translocation, and intestinal epithelial damage in plasma and stool.
	*Duodenal expression of PRR*	Profile the expression of bacterial, fungal, and viral PRR in the duodenal epithelium using immunohistochemistry.
	*Systemic immune cell analysis*	Characterisation of innate and adaptive systemic immune cell phenotypes using differential cell counting and flow cytometry.
	*Plasma and faecal cell cultures*	Evaluation of the immunogenic effect of PAMPs present in plasma and stool on epithelial barrier integrity and function using cell culture models.
*Aim 5*	*Antibiotic reverse permeation*	A novel technique for the assessment of reverse permeation of intravenously administered benzylpenicillin into the gut.
*Aim 6*	Compare all of the above with histological assessments of mucosal biopsies collected at endoscopy, lactulose/rhamnose testing, and established biomarkers of EE.	

## Materials and analysis

### Study design

This cross-sectional study will be conducted in adults in Zambia using a range of stable isotope labelled amino acids and fluorescent tracers, ^1^^,^^19^ systemic immune assessments, EE biomarker analysis, microbiome and metabolome analysis, enzyme functional capacity, proteomics and metaproteomic analysis, barrier integrity and function capacity analysis using cell lines, antibiotic reverse permeation, duodenal morphometry, and duodenal expression of PRR to identify potential new tools for measuring gut functional capacity in Zambian adults with EE. In a sub-study in Zimbabwean adults, we will explore the extent to which antibiotics leak from systemic circulation into the gut, where they may modulate the microbiota. ^20^ The study will be carried out in Harare, where sampling of caecal luminal fluid collected at colonoscopy will be used to quantify reverse permeation of intravenously administered benzylpenicillin from the systemic circulation into the gut lumen. Future work will focus on refinement and simplification of these techniques to make them suitable for use in children.

### Study population

The initial study will be conducted in 80 adults from a population in Lusaka, Zambia, where our previous studies have demonstrated that EE is virtually ubiquitous in adults and children. A further 20 patients attending for routine colonoscopy (for diagnostic purposes) in Harare, Zimbabwe will be enrolled for the antibiotic permeation study.

### Sample size estimation

As there are no, or very limited, data on these measurements in this population, the study was powered based on previous studies using stable isotope breath tests and permeability studies. Previous data from an enteropathy vs. healthy child cohort and ^13^C-sucrose breath tests ^21^ has suggested that calculating the cumulative ^13^CO _2_ excretion, % relative to the dose, at 90 minutes post-intake best represents the villus integrity and the absorption capacity of only the small intestine. Preliminary data have shown that in a group of adults from Misisi with demonstrable sucrase-isomaltase (SI) expression in biopsies, the mean cumulative % of dose excreted as ^13^CO _2_ at 90 minutes is 20.29% compared to 16.02% in the group without SI expression, with SD of 5.29% – resulting in an effect size (ES) of 0.81. With α=0.05, power =(1-β) = 0.80 we estimate we need 20 samples per group for achieving 80% power with a one-tailed test. We will recruit 30 participants into the definitive studies, allowing for potential dropouts and incomplete sample sets. To estimate sample size for permeability studies we used data on circulating LPS concentrations in stunted children which were mean 481 EU/ml (SD 408) in Lusaka, compared to controls (mean 192, SD 113). This results in an effect size of 0.96, with α=0.05 and power=0.80 and we would require 18 in each group for a two-tailed test. The overall sample size was set at 80 adults to allow for sufficient power to cover a range of studies in adults and children.

### Participant recruitment

Consent will begin with an invitation delivered door-to-door, at which all household members eligible to participate will be invited to group discussions. The study group discussion meetings will include the following:

• Introduction to the study team.
• Introduction to the study rationale and protocol in full; explanation of the study.
• Reading of participant information sheet in Nyanja or Shona; literacy rates in Misisi and Harare are variable, and this is therefore the most appropriate method for conveying this information.
• Benefits and risks of being involved in the research.
• Previous participants’ descriptions of being involved in research which includes endoscopy.
• Questions to previous participants.
• Questions to the study team regarding the study.
• Invitation to attend a guided tour of the research and endoscopy facilities.

Potential participants who are unable to make any of the focus group meetings will be invited to the field clinic to discuss the information sheet and study procedures with a member of the study team. All potential participants will be invited to provide written informed consent in individual interviews with a member of the study team afterwards. They will be invited to ask further questions or express concerns. If happy to proceed, they will be asked to sign or thumbprint the consent form, which will be read out to them in the language of their choice if they are unable to read. Consenting and enrolment will occur at least 48 hours after they have attended a focus group meeting or study explanation visit. Only participants who give consent will undergo screening and enrolment into the study. However, if for any reason during the study, the participant decides to withdraw, they will be allowed to exit the study but will not be replaced.

### Inclusion and exclusion criteria

This study will include and exclude participants following the criteria shown in Table 2.

**Table 2.** Inclusion and exclusion criteria.

Inclusion criteria	Exclusion criteria
• In Lusaka, only residents of Misisi compound will be included.	• This study will not include any individuals taking medication for type II diabetes.
• In Harare, only patients from high socio-economic status (i.e., living in detached houses with indoor flush toilets, tiled floors, separate kitchens with running water and indoor stoves, and own at least one vehicle) will be included.	• Any contra-indication to study procedures including endoscopy, such as likely to cause problems with sedation (e.g., oropharyngeal anomaly, previous adverse reaction) or biopsy (e.g., bleeding diathesis).
• Participants of either sex will be included in the study.	• Pregnant or lactating.
• Participants aged between 18 and 65 years old.	• Currently taking any anticoagulants.
• Only participants who are able and willing to undergo HIV testing.	• Having renal failure or liver failure (any major organ system).
• Able and willing to give written, informed consent will be included.	• Any underlying condition, other than HIV, which in the opinion of the investigator would put the subject at undue risk of failing study completion or would interfere with analysis of study results.
	• Participants will be temporarily excluded from study procedures if they have had diarrhoea (by self-report) in the preceding month, and have taken antibiotics, non-steroidal anti-inflammatory drugs, and/or proton pump inhibitors in the preceding month until disqualifying condition has elapsed.

## Protocol

### Screening and initial assessment

Participants will be consented for HIV testing as part of the consent process, before enrolment. Consenting for HIV testing and communication of results will be performed by an experienced and fully trained team of research staff and counsellors. HIV counselling and testing have been performed by this team both as part of standard medical care in the Misisi clinic and as part of the routine research process in our recent studies for over 15 years. ^10^^,^^22^ Likewise, HIV counselling and testing are standard of care in Zimbabwean health care facilities. HIV test results will be given in person and confidence in the clinic of recruitment. Participants found to be HIV seropositive will be allowed to discuss their results and management in more detail. With their consent, they will be referred to HIV specialists for further investigation and management including anti-retroviral therapy per Ministry of Health guidelines and procedures in both Zambia and Zimbabwe. Test results remain confidential and will not be shared with anyone outside of the study team without the participant’s explicit consent.

### Anthropometry

All anthropometry measures will be taken by qualified research nurses trained in anthropometry. Weight will be measured using a calibrated scale, standing height using a height scale, and mid-upper arm circumference (MUAC) using a MUAC tape. Grip strength will be measured using a Takei Grip-D dynamometer, waist and hip circumferences using a measuring tape, and lean and fat mass using a BodyStat ^®^ 1500 Impedance instrument (BodyStat ^®^, Douglas, Isle of Man).

### Dietary assessment

A multi-pass 24-hour dietary recall will be used to assess participants on day 2. All participants will be asked about the food they may have had in the last 24 hours which will be documented (Described in detail elsewhere. ^23^ While this 24-hour period may not be representative of the participants’ habitual intake, it was intended primarily to help interpretation of the metabolic profiling and thus would be most useful prior to the collection of samples for metabolic analysis.

## Clinical investigations: Zambia

**Figure 2.**
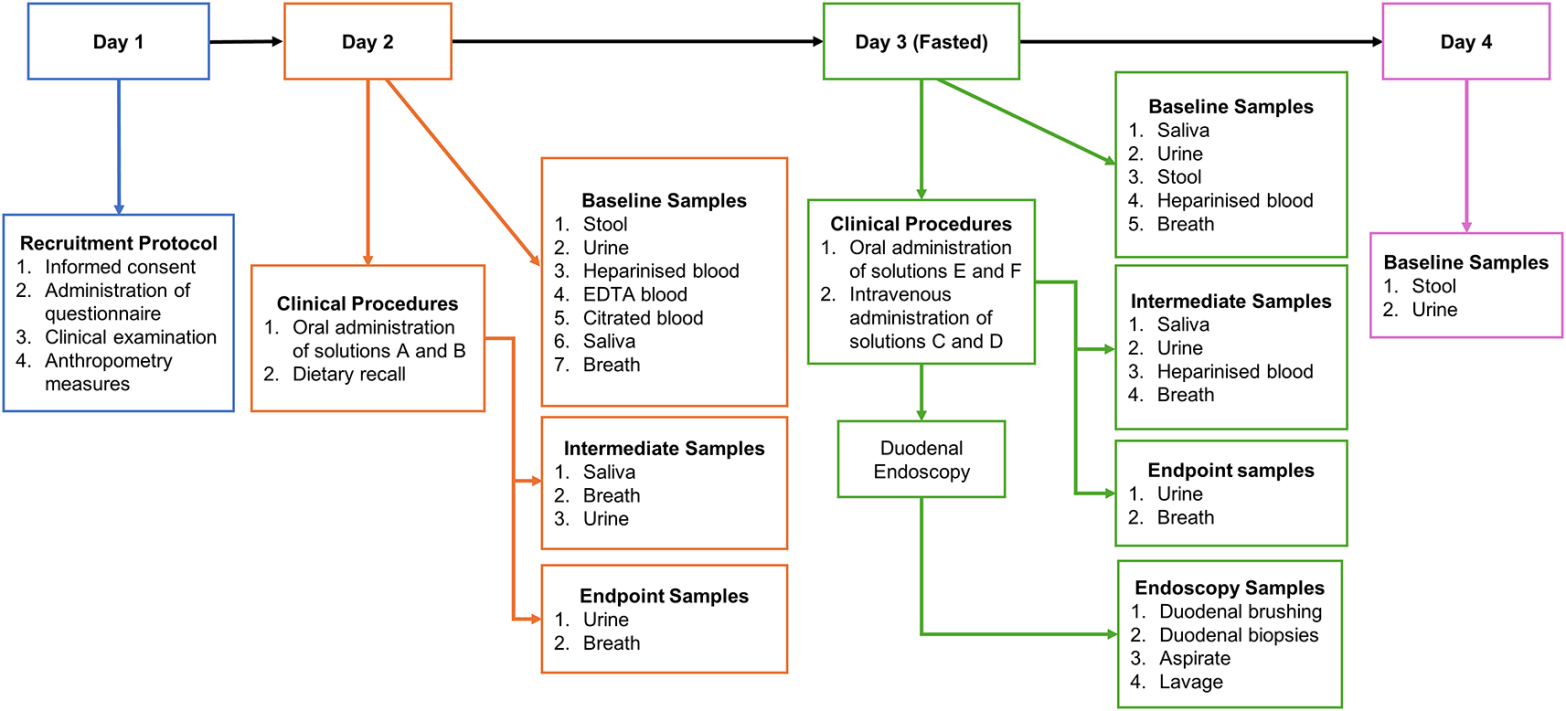
Study schema illustrating sample collection across the 4-day study period. Schema showing each sample collection time point throughout study, Day 1; No samples will be collected; Day 2; 9 mL heparinised blood; 2 mL EDTA blood; 5 mL citrated blood; 11 mL Urine at 00 and 180 mins; 4-8 g of stool; 0.5-2 mL of saliva at 00 and 180 mins; and breath samples every 20 mins. Day 3: 9 mL of heparinised blood; intermediate 2 mL heparinised blood samples every 20 mins for 1 h and every 30 mins for the last 2 h; breath samples every 20 mins; 0.5-2mL of saliva at 00, 60, 120 and 180 mins; 7 mL of urine at 00 and 180 mins; 4-8 g stool; 8 duodenal biopsies and 1 duodenal brushing; Day 4; 2-4 g of stool and 11 mL of urine.

Investigations will be carried out over a four-day period (shown in Figure 2).

### Day 1

This will include the final consent interview, administration of a questionnaire by the study nurse, clinical examination, and anthropometry (including weight, height, impedance, and deuterium approaches to body composition). Urine and stool collection devices will also be given.

### Day 2

Participants will bring urine and stool samples with them. Before administration of the solutions, a saliva sample, 9 mL of blood will be collected in lithium heparin, 2 mL in EDTA and 4.5 mL in citrated blood collection tubes, respectively, by venepuncture. A drink (prepared as shown in Table 3) of ^2^H _2_O (deuterated water, 1 g ^2^H _2_O per kg body weight) containing ^13^C-spirulina protein (1.25 mg kg ^−1^) and ( ^2^H _8_)-Phe (0.035 mg kg ^−1^ in 200 mL clean water) will then be given at, or close to, 08:00 followed by breath sample collection using a straw and Exetainer tube (Labco, UK) every 20 mins for the first 1 h and every 30 mins for the remaining 3 h. A saliva sample will be collected at 180 mins, and a urine sample collection at 180 mins (3 h). A second drink of ^2^H _2_O (1 g ^2^H _2_O per kg body weight) will be given at 180 mins (3 h). During these procedures, a multi-pass 24-hour dietary recall will be administered by the study nurse. At the end of the procedures, the participant will be asked to fast after midnight and given stool and urine collection devices in readiness for day 3 procedures.

**Table 3.** Composition of test solutions.

Solution	Composition	Administration
Solution A	• Deuterated water (D _2_O) (1 mL per kg body weight), • ^13^C-spirulina protein (1.25 mg kg ^−1^), and • ( ^2^H _8_)-Phe (200 mg) in 200 mL water	Oral, given at the beginning of day 2 procedures
Solution B	• Deuterated water (D _2_O) (1 mL per kg body weight)	Oral, given at the end of day 2 procedures
Solution C	• ^2^H _5_-Phe (0.6 μmol kg ^−1^), • 5,5,5- ^2^H _3_-Leu (1.2 μmol kg ^−1^) made up to 10 mL with sterile filtered normal saline	Day 3, given intravenously as slow bolus at beginning of procedures
Solution D	• ^2^H _5_-Phe (0.15 μmol kg ^−1^) • 5,5,5- ^2^H _3_-leucine (0.3 μmol kg ^−1^) made up to 20 mL in sterile filtered normal saline	Day 3, given intravenously as continuous infusion over 3 hours
Solution E	• ^13^C _6_-Phe (0.6 μmol kg ^−1^), • ^13^C _6_-Leu (1.2 μmol kg ^−1^), • 5 g lactulose, • 1 g rhamnose, • 0.5 g D-xylose, • 0.2 g 3-O-methyl D-glucose, • 200 mg sodium fluorescein, • 1.667 mg L-arginine-(guanidineimino- ^15^N _2_) HCl, • 1.0 g glycyl sarcosine in 200 mL water	Day 3, given orally as bolus at beginning of procedures
Solution F	• ^13^C _6_-Phe (0.15 μmol kg ^−1^) • ^13^C _6_-Leu (0.3 μmol kg ^−1^) made up to 600 mL in clean water	Day 3, given orally as sips every 20 mins

### Day 3

Following an overnight fast, an intravenous cannula will be introduced into both arms and 5 mL of blood will be collected into a lithium heparin tube via cannula. The fingertip probe will be clamped gently onto a fingertip, and continuous fluorescence recordings will begin. An intravenous bolus of ^2^H _5_-phenylalanine (0.6 mmol kg ^−1^) and 5,5,5- ^2^H _3_-leucine (1.2 mmol kg ^−1^) in 10 mL normal saline filtered through a 0.2 mm syringe filter (solution C) will be given over 2 mins, and an oral bolus of ^13^C _6_-leucine (1.2 mmol kg ^−1^), ^13^C _6_-phenylalanine (0.6 mmol kg ^−1^), 5 g lactulose, 1 g rhamnose, 0.5 g D-xylose, 0.2 g 3-O methyl-D-glucose, 200 mg fluorescein, 1.667 mg L-arginine-(guanidineimino- ^15^N _2_) HCl and 1.0 g glycyl sarcosine (solution E) given. A continuous intravenous infusion of ^2^H _5_-phenylalanine (0.15 mmol kg ^−1^) and ^2^H _3_-leucine (0.3 mmol kg ^−1^) (solution D) will be commenced, at 5 mL h ^−1^, and sips of ^13^C _6_-phenylalanine (0.15 mmol kg ^−1^) and ^13^C _6_-leucine (0.3 mmol kg ^−1^) (solution F) administered as sips every 20 mins (prepared as shown in Table 3). Breath samples will be collected every 20 mins for the first 1 h and every 30 mins for the remaining 3 h. Blood samples will be collected into lithium heparin collection tubes via the intravenous cannula: 2 mL every 20 mins for the first 1 h and every 30 mins for the remaining 3 h, and 5mL for the last sample. Urine will be collected up to 180 mins (3 h) while saliva collected every 60 mins for 3 h. After 240 mins (4 h) the participant will be transferred to the endoscopy room where upper gastrointestinal endoscopy will be performed using doses of sedation with midazolam and pethidine selected by the endoscopist. Oximetry will be used throughout the procedure. During endoscopy, up to 10 mL of duodenal aspirate will be collected with clean tubing. Using the same tubing, 20 mL 0.9% of sterile saline will be used to collect duodenal lavage. A brushing will be collected using an endoscopic cytology brush, and then the brush will be cut off into 1000 μL PBS. For histology, 3 biopsies will be collected into normal saline using 2.8 mm biopsy forceps, orientated on cellulose acetate paper (Sartorius, Gottingen, Germany) in the endoscopy room and placed in formalin thereafter. An additional 5 biopsies will be collected into 3 cryovials (2+2+1). All samples collected during endoscopy, except for the 3 histology biopsies, will be snap-frozen in liquid nitrogen immediately after collection. At the end of day 3, the participant will be given urine and stool collection devices for the next day.

### Day 4

Only stool and urine samples will be collected.

## Colonoscopy: zimbabwe

An intravenous injection of a single dose of 600 mg benzylpenicillin will be given in patients presenting for colonoscopy, 10 – 15 minutes before the procedure. Luminal fluid from the right side of the colon will be aspirated between 20 and 40 mins after benzylpenicillin administration. This fluid will be centrifuged, stored, and shipped to Imperial College where the assays for the presence of benzylpenicillin, which will indicate reverse permeation, will be performed.

## Sample handling

Samples for safety analysis (differential blood count using the Sysmex XP300™ automated haematology analyser (Sysmex UK Ltd., Milton Keynes, UK) and prothrombin time using the HumaClot Junior (HUMAN Diagnostics Worldwide, Wiesbaden, Germany)) will be processed immediately; excess samples will be retained as backup, but destroyed once the study is complete. Samples for research assays ( Table 4) will be analysed immediately or stored at appropriate temperatures (usually -80°C). Blood samples from Day 2 (9 mL) will be centrifuged (2,300 *g* for 5 minutes at 4°C) to separate plasma from buffy coat cells (enriched for immune cells) and red blood cells; plasma will be aliquoted and stored at -80°C and buffy coat cells will be treated to lyse red blood cells, fixed, washed, re-suspended in cell preservation media, and cryopreserved gradually to -80°C using cell freezing containers to maintain cell integrity. On day 3, blood collected at different time intervals in lithium heparin ( Table 4) will be centrifuged under the same conditions to separate plasma which will be aliquoted and stored at -80°C. Stool samples collected on days 2, 3 and 4 will be aliquoted and stored at -80°C. All saliva samples collected on days 2 and 3, all urine and stool samples collected on days 2, 3, and 4 will also be aliquoted and stored at -80°C. All breath samples that will be collected on days 2 and 3 will be stored at room temperature until analysis. All cryo-stored samples will be retained until batch analyses at either TROPGAN or may be shipped to the relevant external laboratories for analyses (shown in Table 4). For sample types being shipped for analyses at external laboratories, an aliquot of each will be retained at TROPGAN wherever possible; these aliquots will act as a backup in case of analytical failures, or storage failures during transport to another laboratory.

**Table 4.** Summary of laboratory analytical methods.

Sample Type	Assays	Methods	Study participants	Time-points
Stool	Microbiome sequencing	Illumina HiSeq, MinION	All	Baseline
	Barrier integrity and function	Epithelial cell co-cultures		Baseline or Day 2
	Biomarkers	ELISA		Baseline
	Immune signalling assays	Reporter cell line co-culture		Baseline or Day 2
	Dynamic proteomics	LC-MS/MS		Day 3, Day 4
	Metabolic phenotyping	NMR spectroscopy		Day 2, Day 3
Urine	Metabolic phenotyping	NMR spectroscopy	All	Baseline
	Targeted isotope analysis	LC-MS/MS		Day 1, Day 2
Saliva	Body Composition	FTIR	All	Day 1, Day 2
Biopsies	Histology and morphometry	Microscopy	All	Endoscopy samples
	Enzymes and transporters	Immunofluorescence		Endoscopy samples
	Pattern recognition receptor expression	Immunofluorescence, Immunohistochemistry		Endoscopy samples
	Dynamic proteomics	LC-MS/MS		Day 3 samples
Duodenal aspirates	Microbiome sequencing	Illumina HiSeq, MinION	All	Endoscopy samples
	Metabolic phenotyping	NMR spectroscopy		Endoscopy samples
	Dynamic proteomics	LC-MS/MS		Endoscopy samples
	Amino acid uptake in microbiota	LC-MS/MS		Endoscopy samples
	Stable isotope measurement	LC-MS/MS		Endoscopy samples
Plasma	Stable isotope measurement	LC-MS/MS	All	Multiple measurements
	Biomarkers	ELISA, Luminex		Baseline or Day 3
	Barrier integrity and function	Epithelial cell co-cultures		Baseline or Day 3
	Immune function assays	Reporter cell line co-culture		Baseline or Day 3
	Metabolic phenotyping	NMR spectroscopy		Day 2, Day 3
Buffy Coats	Innate and adaptive immune cells	Flow cytometry	All	Baseline
Whole blood	Haematology	Differential cell counts, coagulation	All	Baseline or Day 3
Breath tests	Stable isotope measurement	Infra-red isotope analyser	All	Multiple measurements
Colonic aspirates	Antibiotic assays	LC-MS, HPLC	Aim 3 only	Single aspirates per procedure

ELISA - Enzyme Linked Immunosorbent Assay; LC-MS/MS - liquid chromatography tandem mass spectrometry.

## Clinical assessments

### Permeability analysis

The permeability will be measured by the appearance in the blood of fluorescein ingested orally. Fluorescein has a similar molecular size to lactulose. We have previously demonstrated that the rate and degree of permeation of fluorescein vary under different permeability conditions ^24^ and in patients with intestinal disorders. ^25^ Importantly, following permeation into the blood, fluorescein fluorescence is quantifiable using a fingertip probe similar to a pulse oximeter probe. ^26^

## Laboratory assessments

### Stable isotopes analysis

The ***non-radioactive*** stable isotope labelled amino acids are used to trace the digestion and absorption of protein (day 2) and bidirectional transmucosal amino acid flux (day 3). The labelling strategy is chosen to minimise isotopic crosstalk in the analysis and maximise the metabolic pools that can be sampled ethically. These include breath ( ^13^CO _2_), plasma ( ^13^C / ^2^H amino acids), intestinal aspirates ( ^13^C / ^2^H amino acids), and urine ( ^13^C / ^2^H amino acids). Breath samples will be analysed using a Thermo Scientific™ Delta Ray™ Isotope Ratio Infrared Spectrometer (Thermo Fisher Scientific, Bremen, Germany) in the TROPGAN laboratory (breath ^13^CO _2_) and ^13^C & ^2^H labelled amino acids analysed by a Agilent 1290 Infinity LC coupled to a 6560 triple quadrupole mass spectrometer (Agilent Technologies Inc., Cheadle, UK) in SUERC ( ^13^C/ ^2^H amino acid isotopologues).

### Metabolic phenotyping

Urine, stool, plasma, and duodenal aspirates will be analysed by Nuclear Magnetic Resonance spectroscopy (NMR). High-throughput ^1^H NMR spectra will be acquired using a 600MHz Bruker Avance III™ HD NMR spectrometer equipped with a 5 mm BBI Z-gradient probe, high-order shims, and automated tuning and matching (Bruker Biospin, Rheinstetten, Germany). Samples will be analysed in automation using standard pulse sequences with water suppression. For urine, stool, and duodenal aspirates, two experiments will be acquired: (1D) ^1^H NMR (noesygppr1d, standard Bruker pulse program), and (2D) ^1^H− ^1^H *J*-resolved ( *J*-Res) experiment (jresgpprqf). (1D) ^1^H CPMG (Carr–Purcell–Meiboom–Gill, cpmgpr1d) will also be acquired for plasma only. Each 1D spectrum will be automatically phased and baseline corrected, digitized, and imported into MATLAB for preprocessing and statistical modelling.

### Microbiome analysis

Stool samples, gastric and duodenal aspirates and intestinal biopsies will undergo nucleic acid extraction, library construction and whole metagenome shotgun sequencing at Imperial College London to identify taxonomic and functional composition of multi-site gastrointestinal microbiomes comparing standard short-read sequencing approaches (Illumina HiSeq™ (Illumina, San Diego, CA, USA)) with novel long-read approaches (MinION (Oxford Nanopore Technologies Ltd., Oxford, UK) and PacBio Sequel ^®^ (Pacific Biosciences Inc., Menlo Park, CA, USA)). These sequencing efforts will be duplicated in parallel work in Lusaka to determine the ease of application to an African laboratory. Additional characterisation of microbial uptake of amino acids and conversion of L to D amino acids will also be carried out. Raw sequencing data will be analysed via established bioinformatics pipelines including DADA2 (for 16S rRNA genes), EPI2ME and MetaPhlAn3 (compositional), HUMAnN3 (functional) as with other data, these results will be compared with duodenal biopsy histology, lactulose permeation data, and biomarkers.

### Histology and immunohistochemistry analysis

Formalin fixed biopsies will then be embedded in wax. Sections (4 μm) will be stained using haematoxylin and eosin (H&E) and scanned on an Olympus VS120 ^®^ scanning microscope (Olympus Corporation, Tokyo, Japan). Morphometry will be performed to generate measurements of villus height and crypt depth as has been our standard practice for many years and on which several publications are based. ^8^^,^^27^^,^^28^ Recently a histological scoring system for EE has been developed in which we were involved, and this will be applied to generate scores for villus architectural change, Brunner’s gland penetration, Paneth cell depletion, Goblet cell depletion, intra-epithelial lymphocytosis, and epithelial abnormalities. ^29^ The expression of PRRs will be assessed in separate histological sections of antibody-labelled duodenal biopsies via immunofluorescence microscopy.

### EE biomarker analysis

To characterise the sample sets fully, giving context to observed changes in the composition of the microbiota, biomarkers of EE (shown in Table 5) will be quantified in plasma and stool aliquots via ELISA.

**Table 5.** Plasma and faecal biomarkers of EE.

Marker	Sample type	Measure
1. Endotoxin-core antibody (EndoCab) IgG, IgM, and IgA	Plasma	Microbial translocation
2. Soluble (s)CD14	Plasma	Microbial translocation
3. LPS binding protein (LBP)	Plasma	Microbial translocation
4. sCD163	Plasma	Marker of systemic inflammation
6. C-reactive protein (CRP)	Plasma	Microbial translocation
7. Acid glycoprotein (AGP)	Plasma	Marker of systemic inflammation
8. Myeloperoxidase (MPO)	Stool	Intestinal Inflammation
9. Intestinal fatty acid binding protein (iFABP)	Plasma	Marker of epithelial damage

### Cell culture analysis

To evaluate the capacity of circulating microbial products in plasma and microbial content in stool to activate innate immune signalling (immunogenicity), plasma and faecal water aliquots from each participant will be co-cultured with reporter cell lines (e.g. HEK293, THP-1) for innate immune cell signalling activity. We will add aliquots of plasma/fecal water to the basal/apical side of *in vitro* transwell models of the gut epithelial barrier to evaluate their effect on barrier integrity, permeability, PRR expression, and cellular activation.

### Antibiotic analysis

In the 20 patients attending for routine colonoscopy (for diagnostic purposes) in Harare, using luminal fluid, antibiotic analysis will be carried out by NMR or SUERC by LC-MS.

### Statistical analysis plan

With the complexity of the data set from the techniques described above, the biostatistical framework will be generated in a data-dependent manner. Data from novel tools will be integrated with mucosal morphometry, lactulose permeation, and biomarkers in blood and stool. All data will be tested for normality using a Shapiro-Wilk test and transformed accordingly (normalisation, scaling, etc.). Both parametric and non-parametric tests will be used and both univariate and multivariate analysis will be performed on all datasets generated, as appropriate. For univariate tests, to control for type-I errors, multiple testing will be corrected for by applying the Hommel correction (for Family-Wise Error Rate) or Benjamini-Hochberg (for False Discovery Rate) as appropriate for mildly (positively) correlated variables, or Benjamini-Yekutieli or Storey-Tibshirani (both for False Discovery Rate) methods for highly (positively and inversely) correlated variables. For multivariate tests, data will first be split into training (modelling) and test (evaluation) sets prior to any procedures on the data such as centering, scaling, or other transformations. The predictive classification models will be calculated only on the training portions and evaluated (accuracy and/or F1-score) on the test portions, with hyperparameter tuning using cross-validation on the training data. When small sample sizes do not allow setting aside a completely independent test set, repeated cross-validation will be used to ensure the robustness of the models by calculating a multitude of models (e.g. 1,000 individual models). The evaluation of performance is estimated across all models on the portion of the data that was set aside (test) in each partition. Where algorithms use random initializations of parameters, for each analysis the starting random number seed is saved to allow replication. All data generated will be compared with all duodenal morphometry data which is the most direct measure of EE severity; these comparisons will allow for validation of new real-team assessment tools and established laboratory techniques for sensitivity to differences in EE severity within the cohort.

### Public involvement

Dissemination will include the participants and their families, together with Community Advisory Boards, the University of Zambia School of Medicine, and the University of Zimbabwe Faculty of Medicine and Health Sciences. We will also disseminate results to the Zambian National Health Research Authority and the Medical Research Council of Zimbabwe, and the relevant ethics committees in all the African partner countries, both in written form and by taking opportunities of local research dissemination meetings. This is established practice in all the research groups who will be collaborating on the work proposed. All publications will be open access.

### Data management

Data will be stored on desktop computers until data cleaning is complete, after which locked databases will be uploaded onto secure, password-protected cloud-based systems. Only authorised personnel will have access to the data. All databases will be archived with related questionnaires and any related dictionaries in soft copy on the same drive(s). Long term storage will be on secure cloud-based systems hosted at the Sponsor, QMUL. All data records and materials will be archived appropriately (in accordance with ICH GCP Guidelines) and retained for 5 years according to the Sponsor’s requirements. Data with identifiers will not be stored, for example, personal identifiers on the front sheet of all clinical trial records will be detached prior to archiving and will not be entered on any database. These identifiers will be kept as hard-copy files in locked cabinets in study offices.

### Confidentiality

Data security and confidentiality will be in line with General Data Protection Regulations, HRA Guidance, International Conference on Harmonisation, Guideline for Good Clinical Practice E6, Revision 2, and Queen Mary University of London Data Protection principles and policies. Only authorised study personnel will have access to study files and data. Any data that will be shared with other workers in the field will be anonymised. Identifiable personal data will not be used during analysis or when publishing results. The information that is collected will be treated confidentially. The information entered on separate forms only contains a participant number and the initials and only the data considered important for the study. Research staff are aware of confidentiality of personal data and meeting the requirements of the General Data Protection Regulation (GDPR). Personal information will only be used to contact the patient via letter or telephone after informed consent has been obtained. The only link between personalised data and anonymised data will be a register kept in hard copy in a locked cupboard by the compliance manager. Data will be maintained at an appropriate vigilance (RAID 1) level following current institutional advice. Data access will be restricted to only authorised personnel with individual passwords.

### Ethical considerations

The protocol has been approved by the National Heath Research Agency (REF NHREB00001/02/2022, dated 2 ^nd^ February 2022) and the University of Zambia Biomedical Research Ethics Committee (reference number 2291-2021, dated 20 ^th^ December 2021). The Zimbabwean studies have been approved by the Medical Research Council of Zimbabwe (MRCZ/A/3065, dated 31 July 2023). The work will be carried out in full concordance with the principles of the Declaration of Helsinki, and all participants will give written consent. Queen Mary Ethics of Research Committee issued a letter of no objection (QMERC22.060, dated 22 ^nd^ February 2020).

## Discussion

Our project will take a holistic approach to understanding the impact of EE on gut functional capacity across the whole gastrointestinal tract, which means developing new tools for assessing multiple domains of physiology. This necessitates an assessment of the structure of the intestine (e.g., reduced villus height and therefore surface area, barrier function), digestive and absorptive capacity (e.g., enzyme activity for protein, fat and carbohydrate digestion, transporter expression for absorption), epithelial and immune responses to pathogens, PAMPs, and the microbiota, and the health of the gut microbiota and its metabolic activity. Through this study, we will be able to identify and select the combination of functional measures that are most sensitive to variations in gut physiology according to EE severity in this adult cohort. We will also be able to optimise protocols for communities, such as Misisi, where EE is the predominant gut phenotype. Such measurements will ultimately enable the design of novel therapeutic feeds which actively promote improved gut functional capacity, providing a novel approach to accelerate sustained rehabilitation, reduce mortality, and feed the superorganism (i.e., the host and microbiome), thereby improving growth in malnourished children. ^14^^,^^17^ The urgent problem which we aim to address in this proposal is the dearth of investigative tools available to measure these pathophysiological domains. These tools will be essential for the effective evaluation of new therapeutic feeds to be trialled in SAM and, critically, the selection of those that promote the healing of underlying defects as well as weight gain. Their application to childhood malnutrition will be highly original especially when linked to a clinical trial addressing patient-centred outcomes. Our research group will go on to conduct longitudinal studies and/or clinical trials to evaluate further the tools we will develop. This will provide a deeper understanding of the changing physiology and will generate essential data to support the findings of the trial. In the scenario where no benefit of a novel therapeutic intervention or feed is shown, our novel tools may provide essential for future refinements of the feeds to target specific domains of gut functional capacity and/or enhance effects on pathways showing positive sub-clinical modifications.

In more general terms, these tools may find applications in patients with other enteropathies such as coeliac disease, or enteropathies consequent on cancer therapy. Objective measures of intestinal dysfunction may also help manage patients with intestinal failure. We believe that the strategy of using stable isotopes, fluorescence probes, and new sequencing tools to measure intestinal digestion and absorption, barrier function, and the function of the microbiome, will remain of great interest for years to come.

## Data Availability

No data are associated with this article. Mendeley Data: GI Tools SPIRIT Checklist, http://doi.org/10.17632/2536bhwx7g.1. ^30^ The project contains the following underlying data:
-
GITools_SPIRIT_Fillable-checklist-July2024.pdf Data are available under the terms of the Creative Commons Zero “No rights reserved” data waiver (CC0 1.0 Public domain dedication). The remaining data in this article consists of bibliographic references, which are included in the References section.

## References

[1] Thompson AJ Bourke CD Robertson RC : Understanding the role of the gut in undernutrition: what can technology tell us? *Gut.* 2021 Jun 8;70(8):1580–1594. 10.1136/gutjnl-2020-323609 34103403 PMC8292602

[2] Murray-Kolb LE Rasmussen ZA Scharf RJ : The MAL-ED cohort study: methods and lessons learned when assessing early child development and caregiving mediators in infants and young children in 8 low- and middle-income countries. *Clin. Infect. Dis.* 2014 Nov 1;14(Suppl 4):S261–S272. 10.1371/journal.pone.0221805 25305296 PMC4204608

[3] Nabukeera-Barungi N Grenov B Lanyero B : Predictors of mortality among hospitalized children with severe acute malnutrition: a prospective study from Uganda. *Pediatr. Res.* 2018 Jul;84(1):92–98. 10.1038/s41390-018-0016-x 29795207

[4] Talbert A Thuo N Karisa J : Diarrhoea complicating severe acute malnutrition in Kenyan children: a prospective descriptive study of risk factors and outcome. *PLoS One.* 2012;7(6):e38321. 10.1371/journal.pone.0038321 22675542 PMC3366921

[5] Bwakura-Dangarembizi M Dumbura C Amadi B : Recovery of children following hospitalisation for complicated severe acute malnutrition. *Matern. Child Nutr.* 2022 Apr;18(2):e13302. 10.1111/mcn.13302 34939325 PMC8932709

[6] Kerac M Bunn J Chagaluka G : Follow-up of post-discharge growth and mortality after treatment for severe acute malnutrition (FuSAM study): a prospective cohort study. *PLoS One.* 2014 Jun 3;9(6):e96030. 10.1371/journal.pone.0096030 24892281 PMC4043484

[7] Sow SO Muhsen K Nasrin D : The Burden of Cryptosporidium Diarrheal Disease among Children < 24 Months of Age in Moderate/High Mortality Regions of Sub-Saharan Africa and South Asia, Utilizing Data from the Global Enteric Multicenter Study (GEMS). *PLoS Negl. Trop. Dis.* 2016 May 24;10(5):e0004729. 10.1371/journal.pntd.0004729 27219054 PMC4878811

[8] Amadi B Zyambo K Chandwe K : Adaptation of the small intestine to microbial enteropathogens in Zambian children with stunting. *Nat. Microbiol.* 2021 Apr;6(4):445–454. 10.1038/s41564-020-00849-w 33589804 PMC8007472

[9] Kummerlowe C Mwakamui S Hughes TK : Single-cell profiling of environmental enteropathy reveals signatures of epithelial remodeling and immune activation. *Sci. Transl. Med.* 2022 Aug 31;14(660):eabi8633. 10.1126/scitranslmed.abi8633 36044598 PMC9594855

[10] Amadi B Besa E Zyambo K : Impaired Barrier Function and Autoantibody Generation in Malnutrition Enteropathy in Zambia. *EBioMedicine.* 2017 Aug;22:191–199. 10.1016/j.ebiom.2017.07.017 28750860 PMC5552244

[11] Jones KD Berkley JA : Severe acute malnutrition and infection. *Paediatr Int Child Health.* 2014 Dec;34(Suppl 1):S1–S29. 10.1179/2046904714Z.000000000218 25475887 PMC4266374

[12] Sturgeon JP Mufukari W Tome J : Risk factors for inpatient mortality among children with severe acute malnutrition in Zimbabwe and Zambia. *Eur. J. Clin. Nutr.* 2023 Sep;77(9):895–904. 10.1038/s41430-023-01320-9 37553508 PMC10473959

[13] Sturgeon JP Tome J Dumbura C : Inflammation and epithelial repair predict mortality, hospital readmission, and growth recovery in complicated severe acute malnutrition. *Sci. Transl. Med.* 2024 Feb 28;16(736):eadh0673. 10.1126/scitranslmed.adh0673 38416844 PMC7615785

[14] Subramanian S Huq S Yatsunenko T : Persistent gut microbiota immaturity in malnourished Bangladeshi children. *Nature.* 2014 Jun 19;510(7505):417–421. 10.1038/nature13421 24896187 PMC4189846

[15] Owino V Ahmed T Freemark M : Environmental Enteric Dysfunction and Growth Failure/Stunting in Global Child Health. *Pediatrics.* 2016 Dec;138(6):e20160641. 10.1542/peds.2016-0641 27940670

[16] Gehrig JL Venkatesh S Chang HW : Effects of microbiota-directed foods in gnotobiotic animals and undernourished children. *Science.* 2019 Jul 12;365(6449):eaau4732. 10.1126/science.aau4732 31296738 PMC6683325

[17] Chen RY Mostafa I Hibberd MC : A Microbiota-Directed Food Intervention for Undernourished Children. *N. Engl. J. Med.* 2021 Apr 22;384(16):1517–1528. 10.1056/NEJMoa2023294 33826814 PMC7993600

[18] Barratt MJ Nuzhat S Ahsan K : Bifidobacterium infantis treatment promotes weight gain in Bangladeshi infants with severe acute malnutrition. *Sci. Transl. Med.* 2022 Apr 13;14(640):eabk1107. 10.1126/scitranslmed.abk1107 35417188 PMC9516695

[19] Dorshow RB Johnson JR Debreczeny MP : Transdermal fluorescence detection of a dual fluorophore system for noninvasive point-of-care gastrointestinal permeability measurement. *Biomed. Opt. Express.* 2019 Sep 13;10(10):5103–5116. 10.1364/BOE.10.005103 31646033 PMC6788606

[20] Kelly P Besa E Zyambo K : Endomicroscopic and Transcriptomic Analysis of Impaired Barrier Function and Malabsorption in Environmental Enteropathy. *PLoS Negl. Trop. Dis.* 2016 Apr 6;10(4):e0004600. 10.1371/journal.pntd.0004600 27050312 PMC4822862

[21] Ritchie BK Brewster DR Davidson GP : 13C-sucrose breath test: novel use of a noninvasive biomarker of environmental gut health. *Pediatrics.* 2009 Aug;124(2):620–626. 10.1542/peds.2008-2257 19581263

[22] Louis-Auguste J Kelly P : Tropical Enteropathies. *Curr. Gastroenterol. Rep.* 2017 Jul;19(7):29. 10.1007/s11894-017-0570-0 28540669 PMC5443857

[23] Jobarteh ML McCrory MA Lo B : Development and Validation of an Objective, Passive Dietary Assessment Method for Estimating Food and Nutrient Intake in Households in Low- and Middle-Income Countries: A Study Protocol. *Curr Dev Nutr.* 2020 Feb 7;4(2):nzaa020. 10.1093/cdn/nzaa020 32099953 PMC7031207

[24] Gan J Monfort SE Avery J : Non-invasive assessment of intestinal permeability in healthy volunteers using transcutaneous fluorescence spectroscopy. *Methods Appl Fluoresc.* 2022 Oct 10;10(4):044014. 10.1088/2050-6120/ac9513 36214388

[25] Mbuki R Chileya S Thompson AJ : Rapid testing of gut permeability using oral fluorescein and confocal laser endomicroscopy in Zambian adults. *Trans. R. Soc. Trop. Med. Hyg.* 2021 Oct 1;115(10):1226–1228. 10.1093/trstmh/trab083 34118155

[26] Sanchez EM Avery J Gan J : Transcutaneous fluorescence spectroscopy: development and characterization of a compact, portable, and fiber-optic sensor. *J. Biomed. Opt.* 2024 Feb;29(2):027003. 10.1117/1.JBO.29.2.027003 38419754 PMC10900991

[27] Mulenga C Sviben S Chandwe K : Epithelial Abnormalities in the Small Intestine of Zambian Children With Stunting. *Front Med (Lausanne).* 2022 Mar 16;9:849677. 10.3389/fmed.2022.849677 35372420 PMC8966729

[28] Louis-Auguste J Greenwald S Simuyandi M : High dose multiple micronutrient supplementation improves villous morphology in environmental enteropathy without HIV enteropathy: results from a double-blind randomised placebo-controlled trial in Zambian adults. *BMC Gastroenterol.* 2014 Jan 15;14:15. 10.1186/1471-230X-14-15 24428805 PMC3897937

[29] Liu TC VanBuskirk K Ali SA : A novel histological index for evaluation of environmental enteric dysfunction identifies geographic-specific features of enteropathy among children with suboptimal growth. *PLoS Negl. Trop. Dis.* 2020 Jan 13;14(1):e0007975. 10.1371/journal.pntd.0007975 31929525 PMC6980693

[30] Weatherill J : GI Tools SPIRIT Checklist. *Mendeley Data.* V1. 10.17632/2536bhwx7g.1

