# Supplementary material for: Novel gastrointestinal tools (GI Tools) for evaluating gut functional capacity in adults with environmental enteropathy in Zambia and Zimbabwe: A cross-sectional study protocol
Source: F1000Res. 2025 Mar 4;13:956. Originally published 2024 Aug 23. [Version 2] doi: 10.12688/f1000research.154471.2 (PMC11920692; doi:10.12688/f1000research.154471.2)
Supplement: Peer review history

# Reviewer report: Reviewer response for version 2

**DOI:** 10.5256/f1000research.178271.r369467

**Published:** 2025-03-18

**Authors:** Pascale Vonaesch

**Affiliations:** 1 University of Lausanne, Lausanne, Switzerland

The authors have clarified many of the unclear points. While the overview in the field is stills somehow limited, I do not have any further comments to make to this article. I wish the authors all the best for the actual study and look forward reading their results in the future.

Is the study design appropriate for the research question?

Yes

Is the rationale for, and objectives of, the study clearly described?

Yes

Are sufficient details of the methods provided to allow replication by others?

Partly

Are the datasets clearly presented in a useable and accessible format?

Not applicable

Reviewer Expertise:

childhood undernutrition, environmental enteropathy, human microbiome, small intestinal microbiome, biomarker analysis, clinical microbiome research in Sub-Saharan Africa and South-East Asia

I confirm that I have read this submission and believe that I have an appropriate level of expertise to confirm that it is of an acceptable scientific standard.

# Reviewer report: Reviewer response for version 2

**DOI:** 10.5256/f1000research.178271.r369466

**Published:** 2025-03-11

**Authors:** Declan McCole

**Affiliations:** 1 University of California Riverside Division of Biomedical Sciences, Riverside, California, USA

The authors have addressed my prior concerns.

Is the study design appropriate for the research question?

Yes

Is the rationale for, and objectives of, the study clearly described?

Yes

Are sufficient details of the methods provided to allow replication by others?

No

Are the datasets clearly presented in a useable and accessible format?

Not applicable

Reviewer Expertise:

Intestinal permeability, epithelial tight junctions, epithelial transport, intestinal inflammation.

I confirm that I have read this submission and believe that I have an appropriate level of expertise to confirm that it is of an acceptable scientific standard.

# Reviewer report: Reviewer response for version 1

**DOI:** 10.5256/f1000research.169506.r317023

**Published:** 2024-11-14

**Authors:** Declan McCole

**Affiliations:** 1 University of California Riverside Division of Biomedical Sciences, Riverside, California, USA

The GI TOOLS project is a cross-sectional study that will recruit adults aged 18-65 years with environmental enteropathy (EE) in Lusaka, Zambia that aims to develop new tools for the measurement of gut functional that can be utilized in future clinical trials. The methodology of this project requires each participant to undergo an assessment of gut functional capacity using novel nearpoint-of-care tools and to provide multiple samples for detailed laboratory analyses. Novel techniques include stable isotope approaches to measuring digestion, absorption, and bidirectional transmucosal amino acid flux, a non-invasive fluorescence tool for real-time evaluation of gut permeability, and assessment of gut luminal access  of intravenous antibiotics. Stool and duodenal microbiome sequencing, metabolome analysis of plasma and intestinal fluids, blood immune cell phenotyping, *in vitro *epithelial barrier models, and duodenal immunohistochemistry will also be used to explore EE in depth. These will be integrated with histology and mucosal morphometry using an established histological scoring system for EE, alongside stool and plasma biomarker analysis. The proposal builds on prior foundational work from this consortium that has tested several of the experimental approaches listed in the current proposal. An important feature of the proposal is that local dissemination of research data in Zambia and Zimbabwe will also be undertaken in written form and in presentations at local meetings. This will be an important means of engaging with local research and public health communities. Overall, this represents a very comprehensive set of approaches to functionally characterize the impact of EE on multiple aspects of gut physiology from permeability to digestion of nutrients. The study has high clinical relevance and if it meets its goals it could not only create a new paradigm for functional verification of EE, but also provide robust readouts that could be tested for efficacy of different dietary and therapeutic interventions to rescue subjects from the damaging effects of EE.

 There are some concerns with the inclusion/exclusion criteria. The preference for inclusion of subjects only from higher socioeconomic backgrounds in the Harare (Zimbabwe) study on i.v. antibiotic pharmaco-distribution requires some explanation and justification.

 What considerations will be made if there is a high rate of avoidance of HIV testing?

 What size (kDa and diameter) of fluorescein will be used as the permeability probe? This should be specifically stated and the source/catalog number of the actual fluorescein to be used should also be provided.

 Since proteomic analysis of intestinal biopsies will be performed, it should be clarified if tight junction protein expression will be examined as altered expression could correlate with functional changes in permeability – with the caveat that this approach will not identify changes in localization of TJ proteins that occur without any change in expression levels.

 In Aim 5, Benzylpenicillin is the iv antibiotic that will be used in this study to identify if i.v. administered antibiotics can access the gut lumen. It is unclear what the goal of this aspect of the study is meant to determine. Do the authors consider antibiotic administration to exacerbate symptoms of EE? With respect to the question of whether i.v. antibiotics can enter the gut lumen, this has already been demonstrated that antibiotics can pass into the gut lumen via biliary secretions, and alter bacterial populations (Xue et al, 2023. [Ref 1]). In addition, no explanation of why this particular antibiotic is being used has been included. It is hard to justify this particular aim (#5) as it will not generate any novel findings and could be considered an unnecessary human subjects intervention.

 A limitation of the study is that samples will not be collected longitudinally to identify if readings are sustained or transient. However, longitudinal assessment is proposed in the Discussion section describing future studies.

 Clarification of the “reporter” characteristics of HEK293, THP-1 cell lines should be provided. Will the group be using modified cells lines expressing actual reporter constructs (i.e. to quantify enzymatic activity) or are these cells just being used as ‘model’ cell lines. Given that changes in gut epithelial physiology are integral to the symptoms of EE, what is the rationale for choosing a kidney epithelial cell line (HEK293) as a model epithelium?

 In vitro experiments assessing the effect of PAMPs in fecal samples on permeability of intestinal epithelial cell cultures, will likely exhibit responses to bacterial LPS and it is unclear how the authors will be able to screen for effects of other PAMPs (in feces) in this system with any degree of specificity.

Is the study design appropriate for the research question?

Yes

Is the rationale for, and objectives of, the study clearly described?

Yes

Are sufficient details of the methods provided to allow replication by others?

No

Are the datasets clearly presented in a useable and accessible format?

Not applicable

Reviewer Expertise:

Intestinal permeability, epithelial tight junctions, epithelial transport, intestinal inflammation.

I confirm that I have read this submission and believe that I have an appropriate level of expertise to confirm that it is of an acceptable scientific standard, however I have significant reservations, as outlined above.

## References

[1] : Assessment of the impact of intravenous antibiotics treatment on gut microbiota in patients: Clinical data from pre-and post-cardiac surgery. *Front Cell Infect Microbiol* .2022;12: 10.3389/fcimb.2022.1043971 1043971 10.3389/fcimb.2022.1043971 36741975 PMC9896080

# Response

**Published:** 2025-02-13

**Authors:** James Weatherill

**Affiliations:** Scottish Universities Environmental Research Centre, East Kilbride, Scotland, UK

We are grateful for affording us the opportunity to improve this manuscript.

 Please find the following specific responses to your comments:

 Comments:

- *There are some concerns with the inclusion/exclusion criteria. The preference for inclusion of subjects only from higher socioeconomic backgrounds in the Harare (Zimbabwe) study on i.v. antibiotic pharmaco-distribution requires some explanation and justification.*

*Response:* This point was also made by Reviewer 1 and has been addressed (specific point 2 above).

- *What considerations will be made if there is a high rate of avoidance of HIV testing?*

*Response:* We consider that HIV testing is required to allow interpretation of data and so those potential participants who decline HIV testing cannot be included. Recruitment will continue until the pre-specified sample size is reached.

- *What size (kDa and diameter) of fluorescein will be used as the permeability probe? This should be specifically stated and the source/catalogue number of the actual fluorescein to be used should also be provided.*

*Response:* We use fluorescein sodium as the non-invasive permeability probe, not fluorescein conjugated to other molecules (e.g. FITC-dextran, FITC-PEG, etc). Fluorescein sodium was purchased from Sigma-Aldrich (catalogue number: 46960-100G-F). Fluorescein sodium has a molecular weight of 376 Da. However, the sodium ions dissociate in solution to leave free fluorescein, which has a molecular weight of 332 Da. This means that the molecular weight of the molecule probing the gut barrier in this assay is 332 Da, which is very close to that of lactulose (342 Da), a molecule that has been used to assess gut barrier integrity for decades. Importantly, most previous studies investigating gut barrier integrity in animals and cells using fluorescence-based techniques have used FITC-dextran’s as fluorescent probe molecules. These have much higher molecular weights than fluorescein (e.g. 4 kDa and above). However, as stated above, the molecular weight of free fluorescein is better suited to study of the gut barrier in humans in vivo (due to its similarity to lactulose). Furthermore, our previous results have demonstrated that fluorescein passes the human gut barrier in vivo to differing degrees under different states of intestinal permeability [http://doi.org/10.1088/2050-6120/ac9513, https://doi.org/10.1093/trstmh/trab083] and that orally ingested fluorescein is detectable using non-invasive, transcutaneous spectroscopy while FITC-dextran (4kDa) is not [https://doi.org/10.1038/s41598-020-73149-2]. Thus, taken together, this demonstrates that fluorescein is a suitable fluorescent probe molecule for non-invasive assessment of human gut barrier function.

 Finally, we note that we also use a multi-agent urinary recovery assay for permeability analysis. Using this approach, urinary recovery of five orally ingested molecules (lactulose, rhamnose, D-xylose, 3-O methyl-D-glucose and glycyl sarcosine) is quantified using mass spectrometry. This permits more traditional permeability assessment, for example through measurement of urinary recovery of lactulose and calculation of the lactulose: rhamnose ratio. We have amended the manuscript on pages 13&14 (under Clinical assessments, Permeability analysis) to clarify these points.

- *Since proteomic analysis of intestinal biopsies will be performed, it should be clarified if tight junction protein expression will be examined as altered expression could correlate with functional changes in permeability – with the caveat that this approach will not identify changes in localization of TJ proteins that occur without any change in expression levels.*

*Response*: The proteomic profiling is intended mainly to inform us about synthesis rates. We anticipate that tight junction proteins will be included in the data, and we aim to be able to estimate synthesis rates of these and other proteins which are central to barrier function (including mucins). Additionally, we aim to quantify tight junction proteins in intestinal biopsies (Zonulin, Claudin, Occluding proteins) and for targeted proteins (e.g. ZO-1, Claudin-4) measure 2H incorporation to determine synthesis rates. This clarification has been made in table 1 on page 28.

- *In Aim 5, Benzylpenicillin is the iv antibiotic that will be used in this study to identify if i.v. administered antibiotics can access the gut lumen. It is unclear what the goal of this aspect of the study is meant to determine. Do the authors consider antibiotic administration to exacerbate symptoms of EE? With respect to the question of whether i.v. antibiotics can enter the gut lumen, this has already been demonstrated that antibiotics can pass into the gut lumen via biliary secretions, and alter bacterial populations (Xue et al, 2023. [Ref 1]). In addition, no explanation of why this particular antibiotic is being used has been included. It is hard to justify this particular aim (#5) as it will not generate any novel findings and could be considered an unnecessary human subjects’ intervention.*

*Response:* Thank you for this interesting point. Although it is now known that antibiotics can enter the gut via biliary secretions (penicillin G included), this paper was published (2023) since the protocol was approved. However, we believe that this question still needs to be answered as the Xue study evaluated the impact of antibiotics in patients undergoing cardiac surgery, which is a very different group to the patients we propose to study, notably because hypoperfusion during surgery is well known to drive bowel ischaemia which causes profound barrier loss. Our work is directed at understanding whether the degree of barrier loss in environmental enteropathy is sufficient to permit leakage of antibiotics into the right colon. Penicillin was selected as it is a narrow-spectrum antibiotic which would not be expected to cause significant microbiome disruption in these healthy volunteers, an ethical concern. We have added this to the paper (page 12).

- *A limitation of the study is that samples will not be collected longitudinally to identify if readings are sustained or transient. However, longitudinal assessment is proposed in the Discussion section describing future studies.*

*Response:* This is an important point. In the last stage of the proposed work, we will generate some short term follow up data. Further funding will be sought to explore longitudinal changes in future studies.

- *Clarification of the “reporter” characteristics of HEK293, THP-1 cell lines should be provided. Will the group be using modified cells lines expressing actual reporter constructs (i.e. to quantify enzymatic activity) or are these cells just being used as ‘model’ cell lines. Given that changes in gut epithelial physiology are integral to the symptoms of EE, what is the rationale for choosing a kidney epithelial cell line (HEK293) as a model epithelium?*

*Response:* We agree that we could have been clearer about the specific use of each type of cell line; the reporter lines (THP-1 and HEK293 sub-sets) will be used to evaluate immunogenicity (PAMP content) of the samples in the first instance with THP-1 Dual reporters for NFkB and IRFs used initially and PRR-specific HEK293 used to explore specific PAMP-PRR signalling pathways. The Caco-2 cells, grown in transwell cultures, will be used to assess how the samples impact epithelial barrier function. We have updated this section with more specific details on the separate assays on page 15-16.

- In vitro experiments assessing the effect of PAMPs in fecal samples on permeability of intestinal epithelial cell cultures, will likely exhibit responses to bacterial LPS and it is unclear how the authors will be able to screen for effects of other PAMPs (in faeces) in this system with any degree of specificity.

*Response:* To disaggregate the effects of the different PAMPs in the stool sample, filtered faecal water, PRR-specific inhibitors, antagonists, and antibodies will be used. This information has been added on page 16. We will also use data from PRR-specific HEK293 reporter assays of the samples to identify both LPS/TLR4-driven and non-LPS/TLR4- mediated immunogenicity.

# Reviewer report: Reviewer response for version 1

**DOI:** 10.5256/f1000research.169506.r326299

**Published:** 2024-10-17

**Authors:** Pascale Vonaesch

**Affiliations:** 1 University of Lausanne, Lausanne, Switzerland

In this article, the authors describe a study to take place in Zambia and Zimbabwe, aiming at assessing for possible biomarkers of environmental enteric disorder (EED). They propose to study this in a group of adults which undergo collection of different biospecimens, including blood and stool samples as well as endoscopy and duodenal sampling, allowing to correlate the potential biomarkers with the gold standard, based on histology of the small intestinal mucosa.

 The study is interesting, well designed and addresses a pressing problem, as to date, there are still no widely accepted, easy to measure biomarkers for EED that clearly delineate the disease and distinguish it from other syndromes/infections affecting the GI tract. The study is very interesting as it assesses for macronutrient digestion and absorption as well as intestinal permeability using advanced techniques *in homo* .

 These analyses are combined with a description of the duodenal microbiota using 16S and shotgun metagenomic sequencing, performed in parallel in Africa as well as in the UK using Nanopore technology.

 The study protocol reads very nicely and is complete in its approach and description.

**Major points:**

 My main concern for the study is the introduction and reference list, which is currently biased towards work from the consortium, leaving out previous work performed on biomarkers in studies performed on children by consortia located in Bangladesh, Pakistan, the Central African Republic and Madagascar. In my opinion, even if the target group is children, adding this previous work would greatly strengthen the point of the study performed and situate it better in the overall research performed so far. It would furthermore also give credit to previous work from other groups on the same/similar subject.

 Furthermore, the description on previous work on the (duodenal) microbiota in undernutrition and EED is very scarce and misses to cite previous work in children in the Bangladeshi cohort (Chen R, et al., 2020 [Ref 1]), the Pakistan cohort (Iqbal et al., mSphere 2024 [Ref 3]) and the Afribiota project (Vonaesch et al., PNAS 2018 & 2022 [Ref 2]). Indeed, these studies were the first to show decompartmentalization of the GI tract (first mentioned in Vonaesch et al., PNAS 2018) in stunted growth and EED and could show first hints that the duodenal microbiota could be causally linked to the EED pathophysiology (Chen R, et al., 2020 [Ref 1], Vonaesch et al., 2022 [Ref 2]). This link is currently missing in the manuscript.

 Furthermore, the authors also do not mention previous work linking EED with decreased lipid absorption (Vonaesch et al., PNAS 2022) and metabolization (Habermann et al., Gastroenterology 2021 [Ref 4]).

 Last, there is also only limited references provided for the characterization of the immune system associated with undernutrition and EED (see. i.e. Andrianamantena et al., 2022 [Ref 5] among others). While I acknowledge this previous work is performed in children and not in adults like in he proposed study, I think that the information should still be given, even the more as this work was likely motivating the analyses proposed in the current study protocol and as the authors stress the fact that the biomarkers might be used in children thereafter.

 In conclusion, I strongly encourage the authors to have a more balanced overview of the current state of work on biomarker discovery, the microbiome and EED beyond work performed by the study team in Zambia and in adults.

*Specific points:*

 In the section on study size estimation, it was not clear to me what the prevalence of sucrase-isomaltase expression is. This information would ease the reader to better understand the overall sample size estimations.

 Regarding the inclusion criteria, it was not clear to me why only patients of high economic status are recruited. Will this not lead to a potential bias? Please explain a bit more in detail, so the rationale behind this choice is more evident to the reader.

 Regarding the consenting and consent withdrawal in the study, it was not clear to me what happens to the data and samples if a patient retracks. Are the data kept and anonymized? Destroyed? This should be clarified.

 Regarding the microbiota analysis, it is not clear to me how the characterization of microbial uptake of AA and the subsequent L to D conversion will be measured. Can this be specified?

 In addition, seen the importance attributed to the absolute abundance of the microbiome in the small intestinal tract (demonstrated i.e. in Chen et al, 2020), I encourage the study team to also assess for bacterial absolute abundance in Aim3 of their proposed study aims.

 In the discussion section, it would have been nice to see a bit better how this research is situated in previous work, what it will bring in addition to what has been done before and what the main novelties of this project are compared to previous studies performed on children and/or adults in other countries around the globe. This would allow the reader to better understand the uniqueness and novelty of this study (which is evident to me, but it would be nice to also more clearly showcase this to readers less familiar with the topic).

**Minor points:**

 Page 10, under the heading Day 4: Should this not read: “Colonoscopy: Zimbabwe” (with a capital Z)?

 Data management: who has access to the study data? Will the Zambian study team have access to all data also if generated in the UK? How will the data been made openly accessible after publication (future data availability statement)? Please specify this in the study protocol.

 Ethical considerations: is there a Nagoya approval needed to export samples from Zambia to the UK? If yes, has this approval been applied for/ been granted? Please provide some information on if and how the Nagoya approval applies to this study.

Is the study design appropriate for the research question?

Yes

Is the rationale for, and objectives of, the study clearly described?

Yes

Are sufficient details of the methods provided to allow replication by others?

Partly

Are the datasets clearly presented in a useable and accessible format?

Not applicable

Reviewer Expertise:

childhood undernutrition, environmental enteropathy, human microbiome, small intestinal microbiome, biomarker analysis, clinical microbiome research in Sub-Saharan Africa and South-East Asia

I confirm that I have read this submission and believe that I have an appropriate level of expertise to confirm that it is of an acceptable scientific standard, however I have significant reservations, as outlined above.

## References

[1] : Duodenal Microbiota in Stunted Undernourished Children with Enteropathy. *New England Journal of Medicine* .2020;383(4) : 10.1056/NEJMoa1916004 321-333 10.1056/NEJMoa1916004 PMC728952432706533

[2] : Stunted children display ectopic small intestinal colonization by oral bacteria, which cause lipid malabsorption in experimental models. *Proceedings of the National Academy of Sciences* .2022;119(41) : 10.1073/pnas.2209589119 10.1073/pnas.2209589119 PMC957309636197997

[3] : A shared group of bacterial taxa in the duodenal microbiota of undernourished Pakistani children with environmental enteric dysfunction. *mSphere* .2024;9(6) : 10.1128/msphere.00196-24 e0019624 10.1128/msphere.00196-24 38742887 PMC11332341

[4] : Mucosal Genomics Implicate Lymphocyte Activation and Lipid Metabolism in Refractory Environmental Enteric Dysfunction. *Gastroenterology* .2021;160(6) : 10.1053/j.gastro.2021.01.221 2055-2071.e0 10.1053/j.gastro.2021.01.221 33524399 PMC8113748

[5] : Changes in Systemic Regulatory T Cells, Effector T Cells, and Monocyte Populations Associated With Early-Life Stunting. *Front Immunol* .2022;13: 10.3389/fimmu.2022.864084 864084 10.3389/fimmu.2022.864084 35720335 PMC9202423

# Response

**Published:** 2025-02-13

**Authors:** James Weatherill

**Affiliations:** Scottish Universities Environmental Research Centre, East Kilbride, Scotland, UK

We are grateful for affording us the opportunity to improve this manuscript.

 Please find the following specific responses to your comments:

 Major points:

- *1) My main concern for the study is the introduction and reference list, which is currently biased towards work from the consortium, leaving out previous work performed on biomarkers in studies performed on children by consortia located in Bangladesh, Pakistan, the Central African Republic, and Madagascar. In my opinion, even if the target group is children, adding this previous work would greatly strengthen the point of the study performed and situate it better in the overall research performed so far. It would furthermore also give credit to previous work from other groups on the same/similar subject.*
- *2) Furthermore, the description on previous work on the (duodenal) microbiota in undernutrition and EED is very scarce and misses to cite previous work in children in the Bangladeshi cohort (Chen R, et al., 2020 [Ref 1]), the Pakistan cohort (Iqbal et al., mSphere 2024 [Ref 3]) and the Afribiota project (Vonaesch et al., PNAS 2018 & 2022 [Ref 2]). Indeed, these studies were the first to show decompartmentalization of the GI tract (first mentioned in Vonaesch et al., PNAS 2018) in stunted growth and EED and could show first hints that the duodenal microbiota could be causally linked to the EED pathophysiology (Chen R, et al., 2020 [Ref 1], Vonaesch et al., 2022 [Ref 2]). This link is currently missing in the manuscript.*

*Response:* We fully accept these points and have revised the Introduction (page 5) substantially to embrace the important contributions of seminal work from Bangladesh, Pakistan, and USA, and from the Afribiota studies.

- *3) Furthermore, the authors also do not mention previous work linking EED with decreased lipid absorption (Vonaesch et al., PNAS 2022) and metabolization (Habermann et al., Gastroenterology 2021 [Ref 4]).* *Response:* Again, we should have included this. These references have now been included (page 5).
- *4) Last, there is also only limited references provided for the characterization of the immune system associated with undernutrition and EED (see. i.e. Andrianamantena et al., 2022 [Ref 5] among others). While I acknowledge this previous work is performed in children and not in adults like in he proposed study, I think that the information should still be given, even the more as this work was likely motivating the analyses proposed in the current study protocol and as the authors stress the fact that the biomarkers might be used in children thereafter.*

*Response:* This point is acknowledged, and the introduction now cites more references characterising EE and malnutrition in children where most studies have been done (page 5).

- *5) In conclusion, I strongly encourage the authors to have a more balanced overview of the current state of work on biomarker discovery, the microbiome and EED beyond work performed by the study team in Zambia and in adults.*

*Response:* We have addressed this and hope these points are satisfactory.

 Specific comments:

- *In the section on study size estimation, it was not clear to me what the prevalence of sucrase-isomaltase expression is. This information would ease the reader to better understand the overall sample size estimations.*

*Response:* These estimates are based on a recent publication which was erroneously omitted. We have now included it (Schillinger *et al*).

- *Regarding the inclusion criteria, it was not clear to me why only patients of high economic status are recruited. Will this not lead to a potential bias? Please explain a bit more in detail, so the rationale behind this choice is more evident to the reader.*

*Response:* This choice is partly based on the demographic of the clinic where this work will be done, but also because we reasoned that if antibiotic leakage into the gut lumen is not detectable with higher SES participants, who have milder EE histologically, then it will not be of significance in people with lower SES. This has been added into the paper (page 8).

- *Regarding the consenting and consent withdrawal in the study, it was not clear to me what happens to the data and samples if a patient retracts. Are the data kept and anonymized? Destroyed? This should be clarified.*

*Response:* In the event of withdrawal, the participant is given the choice whether or not to allow retention of samples and data, or if they wish them to be destroyed. This is now included (page 10).

- *Regarding the microbiota analysis, it is not clear to me how the characterization of microbial uptake of AA and the subsequent L to D conversion will be measured. Can this be specified?*

*Response:* A fuller description has now been included (page 15).

- *In addition, seen the importance attributed to the absolute abundance of the microbiome in the small intestinal tract (demonstrated i.e. in Chen et al, 2020), I encourage the study team to also assess for bacterial absolute abundance in Aim3 of their proposed study aims.*

*Response:* This helpful suggestion has been added (pages 14&15).

- *In the discussion section, it would have been nice to see a bit better how this research is situated in previous work, what it will bring in addition to what has been done before and what the main novelties of this project are compared to previous studies performed on children and/or adults in other countries around the globe. This would allow the reader to better understand the uniqueness and novelty of this study (which is evident to me, but it would be nice to also more clearly showcase this to readers less familiar with the topic).*

*Response:* This point was well made. We have expanded the Discussion to include this (pages 19&20).

 Minor points:

- *Page 10, under the heading Day 4: Should this not read: “Colonoscopy: Zimbabwe” (with a capital Z)?*

*Response:* Yes, now corrected (now page 12).

- *Data management: who has access to the study data? Will the Zambian study team have access to all data also if generated in the UK? How will the data been made openly accessible after publication (future data availability statement)? Please specify this in the study protocol.*

*Response:* In compliance with the policies of UKRI, the funder, all data will be open access in a fully anonymised form so that it is fully compliant also with GCP and the requirements of the University of Zambia Biomedical Research Ethics Committee and the Joint Research Council of the University of Zimbabwe. We have described this on pages 17,18&26.

- *Ethical considerations: is there a Nagoya approval needed to export samples from Zambia to the UK? If yes, has this approval been applied for/ been granted? Please provide some information on if and how the Nagoya approval applies to this study.*

*Response:* The research work is fully collaborative, governed by transfer agreements which protect all collaborating researchers and ensure that all data and intellectual property are retained by the institutions in the countries of origin. We have taken advice and been informed that our practises are compliant with the Nagoya protocol.
